# Toward glycerol biorefinery: metabolic engineering for the production of biofuels and chemicals from glycerol

**DOI:** 10.1186/s13068-016-0625-8

**Published:** 2016-10-03

**Authors:** Zhen Chen, Dehua Liu

**Affiliations:** 1 Department of Chemical Engineering, Tsinghua University, Beijing, 100084 China; 2 Tsinghua Innovation Center in Dongguan, Dongguan, 523808 China

**Keywords:** Glycerol, Metabolic engineering, Biorefinery, Biofuel, Biochemical

## Abstract

As an inevitable by-product of the biofuel industry, glycerol is becoming an attractive feedstock for biorefinery due to its abundance, low price and high degree of reduction. Converting crude glycerol into value-added products is important to increase the economic viability of the biofuel industry. Metabolic engineering of industrial strains to improve its performance and to enlarge the product spectrum of glycerol biotransformation process is highly important toward glycerol biorefinery. This review focuses on recent metabolic engineering efforts as well as challenges involved in the utilization of glycerol as feedstock for the production of fuels and chemicals, especially for the production of diols, organic acids and biofuels.

## Background

Glycerol is a traditionally valuable compound which is used in a variety of areas, such as foods and beverages, pharmaceuticals, and cosmetics. In recent years, the fast development of biofuel industry has generated a large amount of crude glycerol as a by-product [1]. Approximately, 10 kg of crude glycerol is generated for every 100 kg of biodiesel produced [2]. The bioethanol process also generates glycerol up to 10 % of the total sugar consumed. The excess of crude glycerol produced in the biofuel industry is leading to a dramatic decrease in glycerol price, making it a waste with a disposal cost for many biodiesel plants. Converting crude glycerol into value-added products is an urgent need and also a good opportunity to improve the viability of biofuel economy. So far, both chemical and biological approaches have been explored to convert glycerol into more valuable products [3–5]. Compared to chemical transformation, biological routes bear several advantages, such as higher specificity, higher tolerance to impurity, being more environmentally friendly (e.g., low temperature/pressure) [6]. Compared to other carbon sources (e.g., glucose and xylose), glycerol has higher degree of reduction and thus can generate more reducing equivalents, conferring the ability to produce fuels and reduced chemicals at higher yields [7]. High abundance, low price and high degree of reduction make glycerol an attractive feedstock for biorefinery [8].

There are a number of microorganisms which can metabolize glycerol. However, direct utilization of natural organisms at the industrial level is often limited by the low production rates, titers, or yields. Thus, metabolic engineering is often needed to improve strain performance, including: (a) elimination of transcriptional repression and enzyme feedback inhibition; (b) reduction of undesired by-products; (c) enhancement of building blocks, reducing power or energy supply; (d) expansion of substrate and product portfolio; and (e) enhancement of strain tolerance to inhibitors and environmental stress [9–14]. More recently, the integration of protein engineering, systems biology and synthetic biology into metabolic engineering has extended strain engineering from local modification to system-wide optimization. Powerful omics technologies, such as genomics, transcriptomics, proteomics and fluxomics, have been combined for in-depth understanding of glycerol metabolism and regulation of microorganism at the system level [15–18].

**Fig. 1:**
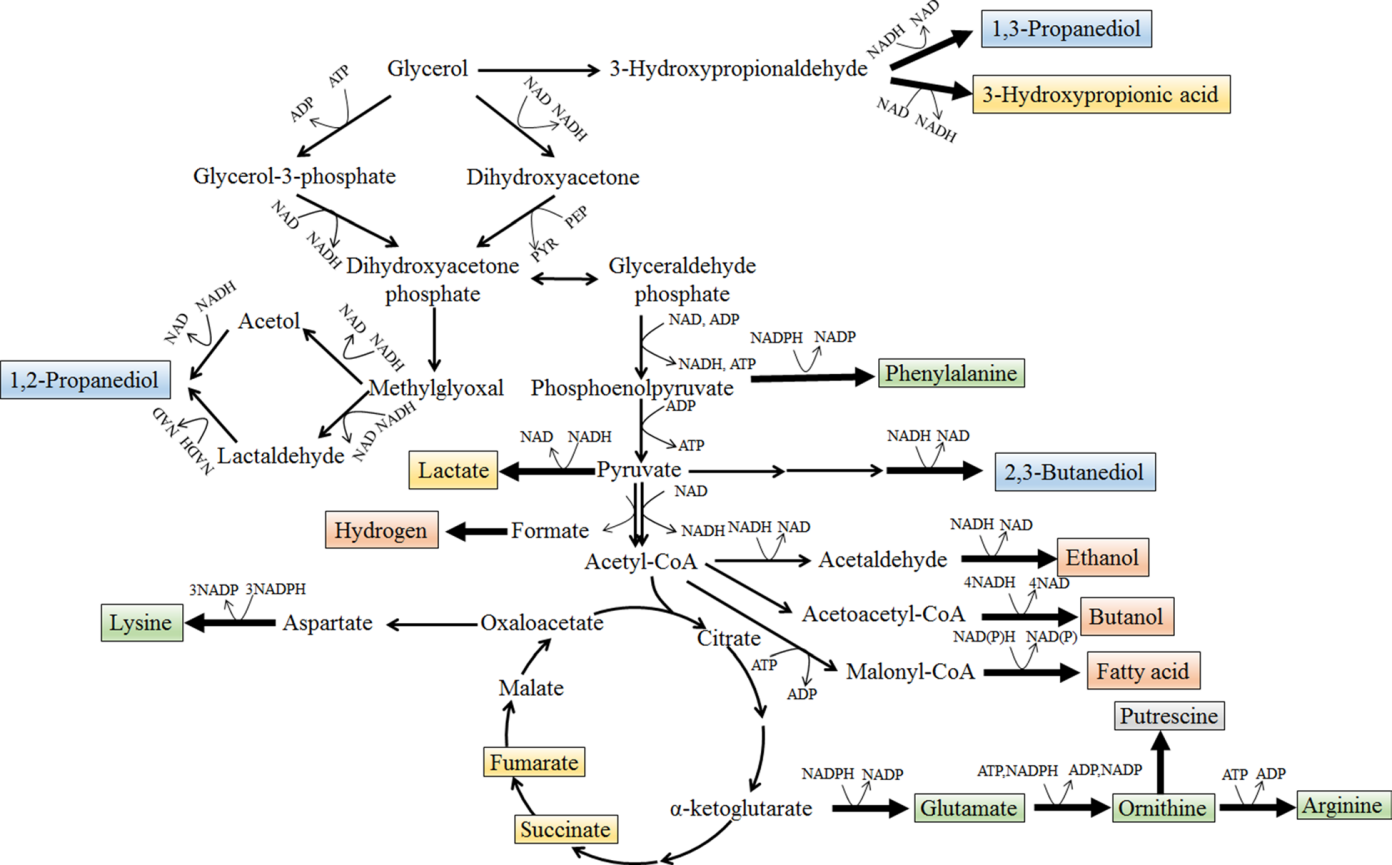
Metabolic pathways of glycerol for the production of various products. Production of diols, amino acids, organic acids, and biofuels via the catabolic pathway of glycerol are illustrated with different colors: diols, *blue*; amino acids, *green*; organic acids, *yellow*; biofuels, *orange*. In some microorganisms, glycerol can be dehydrated to 3-hydroxypropionaldehyde (3-HPA) by glycerol dehydratase. 3-HPA can be converted to 1,3-propanediol by alcohol dehydrogenase or 3-hydroxypropionic acid by aldehyde dehydrogenase. Glycerol can also enter the central metabolic pathway via two routes: (*1*) glycerol kinase and glycerol 3-phosphate dehydrogenase; (*2*) glycerol dehydrogenase and dihydroxyacetone kinase

In general, glycerol is channeled into glycolysis via two metabolic routes (Fig. 1) [7]. In one route, glycerol is dehydrogenated by NAD-dependent glycerol dehydrogenase (*gldA* or *dhaD*) to dihydroxyacetone (DHA), which is then phosphorylated by phosphoenolpyruvate (PEP)- or ATP-dependent dihydroxyacetone kinases (DHAK, *dhaKLM*) to dihydroxyacetone phosphate (DHAP). In the other route, glycerol is first phosphorylated by ATP-dependent glycerol kinase (*glpK*) to glycerol 3-phosphate, and the latter is further reduced by NAD-dependent glycerol 3-phosphate dehydrogenase (*glpD*) to DHAP. The catabolism of glycerol suffers from various genetic and enzymatic regulations. Metabolic control analysis showed that the glycolytic flux during glycerol anaerobic fermentation was almost exclusively controlled by glycerol dehydrogenase and dihydroxyacetone kinase in *Escherichia coli* [19]. Large efforts have been devoted to engineer glycerol catabolism and downstream metabolic pathways for production of various valuable products, including fuels, chemicals, and biomaterials (Table 1). In this review, recent efforts of metabolic engineering to develop industrial strains for glycerol biorefinery are described. Especially, we systematically summarize and discuss different strategies of metabolic engineering for the production of diols, organic acids and biofuels, which represent the most important categories of bulk chemicals in the current biotechnology industry.

**Table 1:** Fermentative production of diols, organic acids, and biofuels from glycerol by metabolic engineered strains

Product and strain	Genetic modification	Titer [g/L]	Yield [g/g]	Culture	References
1,3-Propanediol					
*Klebsiella pneumoniae*	∆*ldhA*	102.06	0.43	Fed-batch	[27]
*Klebsiella oxytoca*	∆*aldA*	70.5	0.58	Fed-batch	[26]
*Clostridium acetobutylicum*	Overexpression of *dhaB1B2* from *C. butyricum* and *yqhD from E. coli*	84	0.51	Fed-batch	[24]
*Lactobacillus reuteri*	Overexpression of *yqhD* from *E. coli*	9.1	0.42	Batch	[22]
*Citrobacter werkmanii*	∆*dhaD*∆*ldhA*∆*adhE*	30.7	0.59	Batch	[23]
*Escherichia coli*	Overexpression of *dhaB1B2* and *dhaT from Clostridium butyricum*	104.4	0.9	Two-stage fed-batch	[34]
1,2-Propanediol					
*Escherichia coli*	∆*ackA*-*pta*∆*ldhA*∆*dhaK*, overexpression *gldA, mgsA, yqhD from E. coli and dhaKL from C. freundii*	5.6	0.21	Batch	[36]
*Escherichia coli*	Overexpression of 1,2-propanediol synthesis enzymes with targeting sequences for inclusion into microcompartments	–	–	Shake flask	[39]
*Saccharomyces cerevisiae*	Overexpression *gldA, mgsA* from *E. coli* and *GUP1, GUT1, GUT2, gdh* from *S. cerevisiae*	2.19	0.27	Batch	[37]
2,3-Butanediol					
* Klebsiella oxytoca*	∆*pduC*∆*ldhA*	131.5	0.44	Fed-batch	[43]
*Bacillus amyloliquefaciens*	Overexpression of *dhaD, acr* and *alsR*	102.3	0.44	Fed-batch	[45]
*Escherichia coli*	Overexpression of *budA and budC*	6.9	0.21	Batch	[44]
l-Lactate					
*Escherichia coli*	∆*pta*∆*adhE*∆*frdA*∆*mgsA*∆*ldhA*∆*lldD*, overexpression *of glpK, glpD* and *ldh*	50	0.89	Shake flask	[50]
*Enterococcus faecalis*	Δ*pflB*	27	0.98	Shake flask	[51]
d-Lactate					
*Klebsiella pneumoniae*	Δ*dhaT*Δ*yqhD*, overexpression of *ldhA*	142.1	0.82	Fed-batch	[46]
*Escherichia coli*	*ΔackA*-*ptaΔppsΔpflBΔdldΔpoxBΔadhEΔfrdA,* overexpression of *ldhA*	100.3	0.75	Fed-batch	[47]
Succinate					
*Escherichia coli*	*ΔackA*-*ptaΔppcΔldhAΔpoxBΔadhE*, overexpression of *pyc*	14	0.69	Shake flask	[53]
*Escherichia coli*	*ΔptsIΔpflB, overexpression of pck*	9.4	1.03	Shake flask	[55]
* Corynebacterium glutamicum*	*ΔackA*-*ptaΔsdhCABΔactΔpqo*, overexpression of *glpFKD*	9.3	0.27	Shake flask	[56]
3-Hydroxypropionic acid					
*Klebsiella pneumoniae*	*ΔdhaTΔyqhD*, overexpression of *puuC*	28	0.39	Fed-batch	[59]
*Escherichia coli*	*ΔackA*-*ptaΔyqhDΔglpR*, overexpression of *dhaB123,gdrAB* and mutant *gabD4*	71.9	–	Fed-batch	[65]
*Pseudomonas denitrificans is*	*Δ3hpdhΔ3hibdhIV*, overexpression of *dhaB123, gdrAB* and *aldH*	3	0.76	Shake flask	[68]
Glutamate					
*Corynebacterium glutamicum*	Overexpression of *glpF, glpK, glpD*	2.2	0.11	Shake flask	[70]
Lysine					
*Corynebacterium glutamicum*	*lysC* ^*P458S*^, *hom* ^*V59A*^, *pyc* ^*T311I*^, *Δpck*, overexpression of *glpF, glpK, glpD*	3.8	0.19	Shake flask	[70]
Ornitine					
*Corynebacterium glutamicum*	*ΔargR, ΔargF*, overexpression of *glpF, glpK, glpD*	2.2	0.11	Shake flask	[71]
Arginine					
*Corynebacterium glutamicum*	*ΔargR*, overexpression of *argB* ^*A26VM31V*^ *, glpF, glpK, glpD*	0.8	0.04	Shake flask	[71]
Putrescine					
*Corynebacterium glutamicum*	*ΔargR,* overexpression of *speC, argF, glpF, glpK, glpD*	0.4	0.02	Shake flask	[71]
Ethanol					
*Escherichia coli*	*ΔfrdABCDΔackA*-*ptaΔldhAΔpoxB*, overexpression of *dhaKLM, gldA* and *adhE*	40.8	0.44	Fed-batch	[76]
Butanol					
*Clostridium pasteurianum*	*ΔdhaT*	8.6	0.26	Shake flask	[78]
Fatty acid					
*Escherichia coli*	*ΔfadDΔfadR*, overexpression of *pntAB, nadK, TE* and *fabZ*	4.82	0.3	Fed-batch	[90]

### Metabolic engineering for the production of diols from glycerol

Diols are compounds with two hydroxyl groups which have a wide range of important applications as chemicals and fuels. The C2–C4 diols are the most important and have been widely used as monomers in the polymer industry. In recent years, microbial production of 1,3-propanediol (1,3-PDO), 1,2-propanediol (1,2-PDO), and 2,3-butanediol (2,3-BDO) from glycerol have been widely investigated (Fig. 1). The production of 1,3-PDO and 2,3-BDO has reached commercial scales.

### 1,3-Propanediol

1,3-PDO is an important diol which has received broad interest in recent years [20, 21]. Of particular interest is its use as a monomer for the synthesis of polyethers, polyurethanes and polyesters such as polytrimethylene terephthalate (PTT). Several species of microorganisms, including *Klebsiella* (*K. pneumoniae* and *K. oxytoca*), *Clostridia* (*C. butyricum* and *C. pasteurianum*), *Enterobacter* (*E. agglomerans*), *Citrobacter* (*C. freundii*) and *Lactobacilli* (*L. brevis* and *L. buchneri*), possess the ability to convert glycerol into 1,3-PDO [22–25]. Among the natural producers, *K. pneumoniae* and *C. butyricum* are the most promising species due to their high tolerance to glycerol inhibition and high production of 1,3-PDO. DuPont has developed a recombinant *E. coli* which can directly utilize glucose for 1,3-PDO production with high titer and yield [21]. This process has been commercialized and considered as a milestone of metabolic engineering. With the dramatic decrease in glycerol price over the past few years, the direct conversion of glycerol to 1,3-PDO is becoming economically competitive to the glucose-based process.

The production of 1,3-PDO from glycerol is a reduction process which consumes 1 mol NADH per mol of 1,3-PDO. In the reductive pathway, glycerol is first dehydrated into 3-hydroxypropionaldehyde (3-HPA) by B12-dependent or B12-independent glycerol dehydratase, and the latter is reduced to 1,3-PDO by alcohol dehydrogenase (Fig. 1). The regeneration of reducing equivalent should be achieved via a glycerol oxidative pathway. Glycerol can be converted into different oxidation products with different yields of reducing equivalent. Under anaerobic condition, oxidation of glycerol to acetate (with formate) is the most effective route for NADH generation with a yield of 2 mol NADH/mol glycerol. Oxidation of glycerol to 2,3-BDO and lactate also generate reducing equivalent with yields of 1.5 mol NADH/mol glycerol and 1 mol NADH/mol glycerol, respectively. Conversion of glycerol to succinate or ethanol, however, does not generate NADH. Thus, coproduction of acetate with 1,3-PDO would give the highest yield of 1,3-PDO under anaerobic condition (0.67 mol/mol). To this end, reduction of succinate and ethanol synthesis could be the first step to channel metabolic flux to more efficient NADH generation pathways. This is proved by Zhang et al. who successfully increased the production of 1,3-PDO by 32.8 % only by knocking out the *aldA* gene to block the synthesis of ethanol by *K. pneumoniae* [26]. Xu et al. attempted to enhance 1,3-PDO production by *K. pneumoniae* by reducing lactate accumulation with the single deletion of d-lactate dehydrogenase gene *ldhA* [27]. Although the formation of lactate was decreased by 95 %, most of the reduced flux in the lactate pathway was not channeled to 1,3-PDO but to the branch of 2,3-BDO, indicating that the flux distribution in the pyruvate node is more flexible than in the glycerol node. Since 2,3-BDO is less toxic to *K. pneumoniae* and synthesis of 2,3-BDO gives higher yield of NADH than lactate, the altered fluxes in the oxidative pathway also benefited 1,3-PDO synthesis, resulting in an increase of 1,3-PDO production by 7 % [27]. Surprisingly, reduction of both lactate and 2,3-BDO formation was reported to be detrimental for glycerol consumption and 1,3-PDO production [28, 29]. Metabolic flux was not channeled to 1,3-PDO or acetate pathway, while high accumulation of pyruvate was observed in the double mutant strain (Δ*ldhA*Δ*budO*), indicating a metabolic imbalance at the pyruvate node [28]. High accumulation of glyceraldehyde 3-phosphate and pyruvate was postulated to be detrimental for 1,3-PDO production, since glyceraldehyde 3-phosphate was strongly inhibitory to glycerol dehydratase [28]. A potential strategy to remove the metabolic imbalance at the PEP/pyruvate node is fine-tuning of downstream enzymes. For example, overexpression of phosphoenolpyruvate carboxylase (*ppc*) and deletion of *arcA* gene, a transcriptional regulator mediating tricarboxylic acid (TCA) cycle flux, were successfully implemented by Dupont to channel the flux from PEP/pyruvate to TCA cycle for by-product’s reduction and NADH regeneration for 1,3-PDO overproduction [21]. Metabolic pathway analysis suggested a maximum yield of 0.84 mol 1,3-PDO/mol glycerol in which only TCA cycle was activated for NADH generation under microaerobic condition [30]. In this case, inactivation of NADH dehydrogenase to block the transfer of electrons from NADH to the quinone pool would be important to increase the NADH pool for 1,3-PDO production.

Minimization of the accumulation of toxic intermediates is another important issue for the high production of 1,3-PDO. 3-HPA is a toxic intermediate during 1,3-PDO synthesis which may cause cell death and fermentation cessation. Reduction of 3-HPA accumulation can be achieved by accelerating the conversion of 3-HPA to 1,3-PDO either by overexpression of 1,3-PDO dehydrogenase (NADH-dependent enzyme encoded by *dhaT* gene or NADPH-dependent enzyme encoded by *yqhD* gene) or by increasing the NADH pool via the modification of fluxes in the oxidation pathway as previously discussed or via the introduction of formate dehydrogenase (*fdh*) [31–33]. Formate dehydrogenase catalyzes the oxidation of formate to carbon dioxide, providing an additional NADH for 3-HPA reduction.

Non-natural producers have also been engineered to produce 1,3-PDO from glycerol. Tang et al. reported the introduction of B12-independent glycerol dehydratase and its activator from *C. butyricum* into *E. coli* in addition to the overexpression of the native alcohol dehydrogenase YqhD [34]. Based on two-stage fermentation (aerobic growth stage and anaerobic production stage), the overall 1,3-PDO yield and productivity reached 104.4 g/L and 2.61 g/L/h at the production stage. Similarly, a recombinant *C. acetobutylicum* was constructed by overexpression of B12-independent glycerol dehydratase and 1,3-PDO dehydrogenase from *C. butyricum*, which can produce 84 g/L of 1,3-PDO with a yield of 0.65 mol/mol [24]. The utilization of B12-independent glycerol dehydratase enhances the economic viability of the designed processes since no expensive B12 is required during the fermentation.

### 1,2-Propanediol

**Fig. 2:**
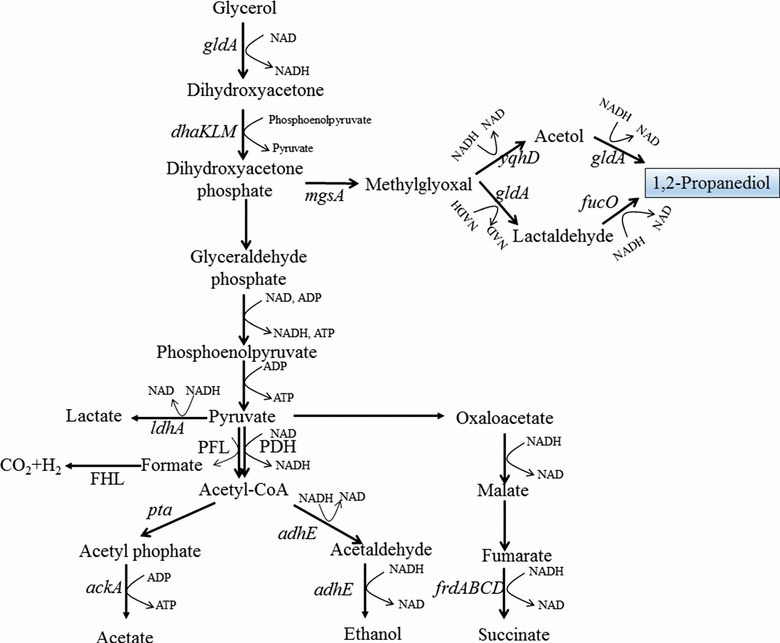
The metabolic pathway for the production of 1,2-propanediol (1,2-PDO). Glycerol is converted to 1,2-PDO via two alternative pathways: one with acetol as an intermediate and the other with lactaldehyde as an intermediate. *ackA* acetate kinase; *adhE* acetaldehyde/alcohol dehydrogenase; *dhaKLM* dihydroxyacetone kinase; *FHL* fomate hydrogen lyase complex; *frdABCD* fumarate reductase; *fucO* 1,2-PDO reductase; *gldA* glycerol dehydrogenase; *ldhA* lactate dehydrogenase; *mgsA* methylglyoxal synthase; *PDH* pyruvate dehydrogenase; *PFL* pyruvate formate-lyase; *pta* phosphate acetyltransferase; *yqhD* alcohol dehydrogenase

1,2-PDO is another important diol with a global market of about 1.5 million tons/year. It is used in a wide spectrum of areas such as: (a) production of polyester resins; (b) antifreezing; (c) detergents; and (d) cosmetics [35]. Several attempts have been made to optimize 1,2-PDO production from glycerol [36, 37]. Similar to 1,3-PDO, the conversion of glycerol to 1,2-PDO is a reduction process which consumes 1 mol NADH per mol of 1,2-PDO (Fig. 2). In the 1,2-PDO synthesis pathway, DHAP is first converted to methylglyoxal (MG) via methylglyoxal synthase (*mgsA*). The conversion of MG into 1,2-PDO takes place through two alternative pathways, one with acetol as an intermediate through the action of alcohol dehydrogenase (*yqhD*) and glycerol dehydrogenase (*gldA*), and the other with lactaldehyde as an intermediate mediated by glycerol dehydrogenase (*gldA*) and 1,2-PDO reductase (*fucO*). Overexpression of either pathway is technologically feasible for 1,2-PDO production [36]. However, to overproduce 1,2-PDO, several issues should be considered. First, glycerol assimilation is coupled with PEP synthesis in *E. coli* since DHAK utilizes PEP as the phosphate donor in the phosphorylation of DHA. Thus, a high flux from DHAP to PEP is required during glycerol assimilation which significantly reduces the precursor availability for 1,2-PDO synthesis. This problem could be overcome by substitution of PEP-dependent DHAK with ATP-dependent DHAK. This was proved to be highly efficient for enhancing 1,2-PDO yield and glycerol consumption in *E. coli* [36]. Second, since 1,2-PDO synthesis is both redox and ATP consuming (with ATP-dependent DHAK), the coproduction of another oxidation product is necessary in the anaerobic condition. As discussed in the previous session, the conversion of glycerol to acetate or lactate generates NADH, while the conversion of glycerol to ethanol (with formate) or succinate is redox neutral. Coupling the production of acetate with 1,2-PDO should give the highest yield of 1,2-PDO. However, this maximum yield is never quite reached because there is no net gain of ATP for cell growth and maintenance. Thus, coproduction of ethanol to provide ATP is considered to be necessary for 1,2-PDO production under anaerobic conditions [36]. Interestingly, it was found that blocking the synthesis of acetate (Δ*ackA*-*pta*) and/or lactate (Δ*ldhA*) increased the production of 1,2-PDO, while disrupting the synthesis of ethanol (Δ*adhE*) or succinate (Δ*frdA*) reduced both glycerol consumption and 1,2-PDO production by *E. coli* under anaerobic conditions [36]. Deletion of ethanol or succinate channeled most of the flux toward acetate or lactate formation, which would cause significant redox imbalance and growth deficiency. On the other hand, it was observed that there was an increase of ethanol production for the mutants with the disruption of acetate and/or lactate production pathways [36]. The conversion of glycerol to ethanol (and formate) is a redox-neutral process with the generation of 1 mol ATP. Thus, the enhanced ethanol production could provide more ATP for cell growth without affecting the redox balance. However, since the ethanol (and formate) pathway does not generate NADH, the accumulation of pyruvate as oxidized product and the activation of pyruvate dehydrogenase and/or formate hydrogen lyase (FHL) were necessary for redox balance in the acetate- (Δ*ackA*-*pta*) and/or lactate-deficient (Δ*ldhA*) mutants [36]. With the deletion of *ackA*-*pta, ldhA,* and PEP-dependent *dhaK* in addition to overexpression of *gldA*, *mgsA*, *yqhD,* and ATP-dependent DHAK (*C. freundii dhaKL*), the mutant *E. coli* can produce 5.6 g/L of 1,2-PDO with a yield of 0.213 g/g glycerol. Similarly, *S. cerevisiae* strains have been engineered to produce 1,2-PDO by modifying both glycerol dissimilation and 1,2-PDO synthesis pathways [37]. Overexpression of glycerol kinase (GUT1), glycerol 3-phosphate dehydrogenase (GUT2), glycerol dehydrogenase (*gdh*), and a glycerol transporter gene (GUP1) increased glycerol utilization and cell growth rate. Further introduction of *mgsA* and *gldA* from *E. coli* enabled the production of 2.19 g/L of 1,2-PDO by the metabolically engineered *S. cerevisiae*.

The conversion of glycerol to 1,2-PDO generates several toxic intermediates such as MG and lactaldehyde. To minimize the accumulation of toxic intermediates, assembly of pathway enzymes by DNA scaffolding and bacterial microcompartment (BMC) have been tried. Conrado et al. utilized fused zinc-finger domains which can specifically bind the DNA scaffold to assemble methylglyoxal synthase, 2,5-diketo-d-gluconic acid reductase and glycerol dehydrogenase in *E. coli* [38]. Under optimized enzyme/scaffold ratio, the production of 1,2-PDO was improved by ~3.5 times. In another attempt to construct artificial BMC for 1,2-PDO synthesis, N-terminal targeting sequences derived from Pdu BMC were added to glycerol dehydrogenase, dihydroxyacetone kinase, methylglyoxal synthase and 1,2-PDO oxidoreductase, resulting in the formation of protein inclusions within the recombinant *E. coli* cell [39]. Interestingly, irrespective of the presence of BMCs, a strain containing the fused enzymes exhibited a 245 % increase of 1,2-PDO production in comparison to the strain with free enzymes. It was observed that the enzymes with targeting peptides formed dense protein aggregates, which may result in increased channeling of substrates and products between proteins due to proximity effects.

### 2,3-Butanediol

2,3-BDO is a potential platform chemical which can be used as biofuel as well as building blocks for the synthesis of 2-butanone [40], 2-butanol [41] and 1,3-butadiene [42]. As discussed before, the conversion of glycerol to 2,3-BDO is an oxidation process with a theoretical yield of 0.5 mol/mol. The NADH generated during the production of 2,3-BDO should be consumed by coupling it with the generation of a reduced product or with oxidative phosphorylation. Thus, aerobic condition is often employed for 2,3-BDO production in which oxygen can be used as electron acceptor. One well-studied case is *K. oxytoca* which can efficiently synthesize both 1,3-PDO and 2,3-BDO from glycerol. Aeration and pH are two critical factors affecting the distribution between 1,3-PDO and 2,3-BDO [43–45]. High aeration and low pH (pH6) are beneficial for 2,3-BDO production, while low aeration and higher pH (pH7) enhance the accumulate of 1,3-PDO. With optimal pH and aeration, a mutant *K. oxytoca* strain blocking the synthesis of 1,3-PDO and lactate (Δ*pduC*Δ*ldhA*) can accumulate 131.5 g/L of 2,3-BDO from crude glycerol, with a productivity and a yield of 0.84 g/L/h and 0.44 g/g. In another study, overexpression of glycerol dehydrogenase, acetoin reductase, and a transcriptional regulator ALsR were combined to enhance the metabolic flux toward 2,3-BDO synthesis in *Bacillus amyloliquefaciens*. The engineered strain showed reduced accumulation of acetoin and other by-products. Similarly, a combination of three-stage dissolved oxygen control strategy (350 rpm for the first 5 h; 400 rpm from 5 to 22 h; and 350 rpm thereafter) and two-stage pH control strategy (without pH control for the first 16 h and with constant pH at 6.5 thereafter) was applied for this mutant during the fermentation, which allowed a high titer (102.3 g/L) and yield (0.44 g/g) of 2,3-BDO production [45]. The high titer and yield in these examples demonstrated that the biological production of 2,3-BDO could be both technologically and economically competitive.

### Metabolic engineering for the production of organic acids from glycerol

Beyond traditional uses in the feed and food industry, recent interest in bioplastics and bulk chemicals has pushed the biological production of organic acids from renewable raw materials. Glycerol has been utilized to produce various organic acids, including lactate, succinate, fumarate, 3-hydroxypropionic acid (3-HP), and amino acids (Fig. 1).

### Lactate

Lactate is an important organic acid with many applications in the food, pharmaceutical, and chemical industries. In particular, the synthesis of biodegradable polylactate (PLA) from d- or l-lactate holds a great potential to substitute for the synthetic plastics produced by the petrochemical industry. Biological production of lactate from glycerol is a promising process with a theoretical yield of 1.0 mol/mol. Similar to 2,3-BDO, biological production of lactate from glycerol is an oxidation process which generates net NADH and ATP. Thus, microaerobic condition is preferred for lactate production from glycerol, while anaerobic fermentation is preferred when glucose is used as substrate. Several strains can natively produce a large amount of lactate under anaerobic and microaerobic condition. For example, d-lactate is produced as a main by-product by *K. pneumoniae* and *E. coli* during glycerol assimilation. With the deletion of *dhaT* and *yqhD* gene to block the synthesis of 1,3-PDO and overexpression of the native d-lactate dehydrogenase gene *ldhA*, *K. pneumoniae* was able to accumulate up to 142.1 g/L of d-lactate with a yield of 0.82 g/g glycerol under microaerobic condition [46]. More systematical strategies have also been applied to *E. coli* for d-lactate production [47–49]. A homolactic strain for d-lactate production from glycerol was constructed by systematic elimination of all by-products synthetic routes, including acetate (Δ*ackA*-*pta*Δ*poxB*), ethanol (Δ*adhE*), succinate (Δ*pps*Δ*frdA*), formate (Δ*pflB*), and d-lactate assimilation (Δ*dld*). The yield of d-lactate was further improved from 0.67 to 0.75 g/g by overexpression of d-lactate dehydrogenase from a low-copy plasmid [47]. Similar strategies can be adopted for l-lactate production [50, 51]. Mazumdar et al. developed a highly efficient l-lactate producer by four steps: (1) replacing the native d-lactate dehydrogenase (Δ*ldhA*) with l-lactate dehydrogenase from *Streptococcus bovis*; (2) inactivating the methylglyoxal pathways (Δ*mgsA*) to avoid the synthesis of lactate racemic mixture; (3) inactivating l-lactate assimilation pathway (Δ*lldD*); and (4) blocking the synthesis of acetate (Δ*pta*), ethanol (Δ*adhE*), and succinate (Δ*frdA*) [50]. The resulting *E. coli* strain produced l-lactate from crude glycerol with high yield (0.89 g/g) and optical purity (99.9 %). Thus, it is highly competitive to produce both d-lactate and l-lactate from cheap crude glycerol as compared to the glucose-based routes.

### Succinate

Succinate is considered as one of the top 12 building block chemicals [52, 53]. It can be used as solvents, pharmaceutical products, or monomer for the synthesis of biodegradable plastics. Transformation of glycerol to succinate is a redox-neutral process. Theoretically, glycerol can be converted into succinate with a yield of 1 mol/mol with the addition of supplementary bicarbonate in the medium for anaplerotic reaction [52]. This yield is much higher than the one observed when utilizing glucose as carbon source. Enhancing anaplerotic flux is considered to be the most important factor for high yield production of succinate. There are three enzymes involved in anaplerotic reactions: phosphoenolpyruvate carboxylase (PEPC), phosphoenolpyruvate carboxykinase (PEPCK), and pyruvate carboxylase (PYC). Overexpression of both PEPC and PYC are shown to be beneficial for succinate production. In most bacteria, PEPC and PYC catalyze the carboxylation reactions, while PEPCK catalyzes the decarboxylation reaction. Reversing the direction of the gluconeogenic PEPCK reaction could generate net ATP during CO_2_ fixation and thus can be an efficient way to enhance succinate production under anaerobic condition. Overexpression of PEPCK together with the increase of the PEP pool is important to reverse the direction of PEPCK. For example, overexpression of the native PEPCK was combined with the deletion of pyruvate formate-lyase (Δ*pflB*) to increase the yield and production of succinate in *E. coli*. Similarly, overexpression of PEPCK from *Actinobacillus succinogenes,* the main CO_2_-fixing enzyme in *A. succinogenes*, increased succinate production 6.5-fold in an *E. coli* mutant when combined with the deletion of PEPC [54]. Overexpression of PEPCK in *E. coli*, together with the disruption of pyruvate formate-lyase (Δ*pflB*) and phosphotransferase system (Δ*ptsI*) to increase the pool of PEP, allowed the conversion of glycerol to succinate with a yield of 0.80 mol/mol under anaerobic condition [55]. This yield is much higher than the strain which overexpressed PYC and suppressed all by-products synthesis (acetate, ethanol, and lactate) [53]. Aerobic succinic acid production from glycerol was realized in *C. glutamicum* by disruption of the TCA cycle downstream of succinate (Δ*sdhCAB*) and suppression of acetate formation (Δ*ackA*-*pta*Δ*act*Δ*pqo*) in addition to overexpression of PYC, PEPC, and glp regulon for glycerol assimilation (*glpFKD*) [56]. Under optimized fermentation conditions, the strain exhibited a yield of 0.21 mol/mol and a titer of 9.3 g/L [56]. From a practical point of view, aerobic production of succinate is not economically reasonable, since it has lower theoretical yield and higher energy requirement as compared to anaerobic condition.

### 3-Hydroxypropionic acid

3-Hydroxypropionic acid (3-HP) is a versatile platform chemical which can be used for the production of acrylic acid, acrylonitrile, malonic acid, and 1,3-PDO [57]. Different metabolic pathways have been predicted and tried for the production of 3-HP from several intermediates including glycerol, lactate, malonyl-CoA, and β-alanine [57]. Direct conversion of glycerol to 3-HP is considered as one of the most promising routes for 3-HP production. Unlike the production of 1,3-PDO, the formation of 3-HP from glycerol is an oxidation process generating net NADH. Thus, it is feasible to coproduce 3-HP with 1,3-PDO to recycle the reducing equivalent. This concept was proved in recombinant *K. pneumoniae* which produced 48.9 g/L of 3-HP and 25.3 g/L of 1,3-PDO by overexpressing an *E. coli* aldehyde dehydrogenase (*aldH*) [58]. On the other hand, 3-HP can be produced as the sole product if an electron acceptor is provided [59]. Under anaerobic condition, nitrate was used as an electron acceptor for 3-HP production by a mutant *K. pneumoniae* with double deletion of *dhaT* and *glpK* to reduce the flux toward 1,3-PDO synthesis and the glycerol oxidation pathway [60]. Under aerobic condition, oxygen can be utilized as an electron acceptor. However, control of the aeration is important for the production of 3-HP. Increasing dissolved oxygen is beneficial for NAD recycling; however, high oxygen concentration also inhibits the synthesis of coenzyme B12 and reduces the activity of glycerol dehydratase. Thus, it is important to combine both metabolic engineering (reduction of 1,3-PDO and other by-products formation) and process optimization for the high production of 3-HP by *K. pneumoniae*.

*Escherichia coli* as a heterologous host has also been engineered to produce 3-HP from glycerol. Similarly, reduction of 1,3-PDO synthesis by *yqhD* deletion is important for increasing the production of 3-HP [61]. Knockout of *glpK* to block glycerol oxidation pathway, however, significantly reduced cell growth and glycerol consumption [62]. Thus, L-arabinose-inducible promoter was used for fine-tuning of the expression of *glpK,* and deletion of *glpR,* a repressor of *glp* regulon, was also combined to enhance glycerol metabolism [62]. Another smart strategy using toggle switch to conditionally repress gene expression has also been tried. By controlling the adding time of IPTG, a toggle switch to simultaneously induce the 3-HP synthesis pathway (glycerol dehydratase and aldehyde dehydrogenase) and repress glyceraldehyde 3-phosphate dehydrogenase could increase 3-HP production compared to the control strain [63]. *In silico* metabolic simulation has been utilized to identify new targets for increasing the yield of 3-HP. A genome-scale modeling identified triosephosphate isomerase (*tpiA*) and glucose 6-phosphate dehydrogenase (*zwf*) as candidates for improving 3HP production [64]. The 3-HP yield of a triple *E. coli* mutant (Δ*tpiA*Δ*zwf*Δ*yqhD*) was increased by 7.4-fold in comparison to the parental strain. Compared to *K. pneumoniae*, the key challenge of using *E. coli* as host for 3-HP production is the need to add expensive coenzyme B12 during fermentation.

Metabolic balance between glycerol dehydratase and aldehyde dehydrogenase to reduce 3-HPA accumulation is also important for 3-HP production. This could be achieved using more active aldehyde dehydrogenase or controlling the expression of glycerol dehydratase [65, 66]. Chu et al. screened a highly active aldehyde dehydrogenase from *Cupriavidus necator* and used site-directed mutagenesis to improve its activity [65]. The most active mutant bears two point mutations: E209Q/E269Q. *Escherichia coli* transformed with this mutant produced up to 71.9 g/L of 3-HP with a productivity of 1.8 g/L/h. Lim et al. used UTR (untranslated region) engineering to precisely control the expression of glycerol dehydrogenase (*dhaB1*) for optimal metabolic balance [67]. It was observed that high expression of glycerol dehydrogenase resulted in low cell growth and high accumulation of 3-HPA. Under optimal expression level of glycerol dehydratase, the best strain produced 40.51 g/L of 3-HP with a yield of 0.97 g/g glycerol (using glycerol and glucose as co-substrates).

Since it is necessary to add the expensive coenzyme B12 during 3-HP production by *E. coli*, *Pseudomonas denitrificans* which can naturally produce coenzyme B12 under aerobic conditions was engineered to produce 3-HP. When glycerol dehydratase and its activator and an aldehyde dehydrogenase from *K. pneumoniae* (*puuC*) were overexpressed, the recombinant strain could produce 4.9 g/L of 3-HP using glycerol and glucose as co-substrates [68]. However, *P. denitrificans* can degrade 3-HP and utilize it as carbon source. Thus, the 3-HP degradation pathway should be identified and deleted [69].

### Amino acids

l-Amino acids are very important fermentation products which have been widely used in industry. Due to the high reduction degree of glycerol, it is very promising to utilize glycerol as substrate for amino acid production, which often consumes a large amount of reducing equivalents. The dominant strain for l-amino acid production, *C. glutamicum,* has been engineered to utilize different substrates including glycerol. Although *C. glutamicum* possesses putative homologs of glycerol kinase (*glpK*) and glycerol-3-phosphate dehydrogenase (*glpD*), wild-type *C. glutamicum* cannot grow with glycerol as the sole carbon source. Glutamate production from glycerol was enabled by plasmid-borne expression of *E. coli glpF*, *glpK*, and *glpD* in *C. glutamicum* with the titer of 2.2 g/L [70]. Introduction of *glpFKD* into *C. glutamicum* strains producing other amino acids led to the synthesis of lysine, ornithine, arginine, phenylalanine, and putrescine from crude glycerol (Table 1) [71, 72]. The titers (0.4–3.8 g/L) and yields (0.02–0.2 g/g), however, are much lower compared to cultures using glucose as carbon source. One of the potential reasons is the low yield of NADPH during glycerol catabolism. Further enhancement could be achieved by introducing novel NADPH generation pathways such as transhydrogenase, NADPH-dependent glycerol dehydrogenase or NADPH-dependent glyceraldehyde phosphate dehydrogenase [73].

### Metabolic engineering for the production of biofuels from glycerol

Due to its low price and high reduction degree, glycerol is a very promising feedstock for biofuel production in comparison to glucose and other sugars. Production of ethanol from glycerol has bene intensively studied, while the research on the transformation of glycerol to butanol and lipids/fatty acids has just started in recent years.

### Ethanol

As discussed before, the transformation of glycerol to ethanol (with formate) is a redox-neutral process with net ATP generation; thus, it is possible to eliminate most by-products formation in anaerobic condition. Specifically, co-production of ethanol-hydrogen or ethanol-formate can be obtained with yield close to 1.0 mol/mol [74]. For example, double mutation of fumarate reductase (Δ*frdA*) and phosphotransacetylase (Δ*pta*) to eliminate succinate and acetate synthesis is enough to engineer an *E. coli* strain producing ethanol and hydrogen at maximum theoretical yield (1.0 mol/mol) at pH 6.3 under anaerobic condition [75]. The final titer and productivity, however, were relatively low although glycerol consumption could be partially strengthened by overexpression of glycerol dehydrogenase (*gldA*) and dihydroxyacetone kinase (*dhaKLM*). Wong et al. recently constructed an inducer- and antibiotic-free system to increase the titer, yield and specific productivity of ethanol under microaerobic condition [76]. To achieve such a goal, the pathways to by-products (succinate, acetate, and lactate) were first inactivated (Δ*frdABCD*Δ*ackA*-*pta*Δ*poxB*Δ*ldhA*). The glycerol uptake genes *dhaKLM*, *gldA*, and *glpK* and the ethanol pathway gene *adhE* were then overexpressed under native *E. coli* promoters in a plasmid. The chromosomal *gldA* and *glpK* were deleted to inactivate the native glycerol consumption pathway. Thus, only the strains bearing a plasmid-borne *gldA* can utilize glycerol as the sole carbon source. This strategy eliminates the need for antibiotics to retain the plasmid during fermentation. The final engineered strain can produce ethanol with high titer and yield (40.8 g/L and 0.44 g/g) without using expensive inducers and antibiotics. This process is very promising for industrial ethanol production.

### Butanol

**Fig. 3:**
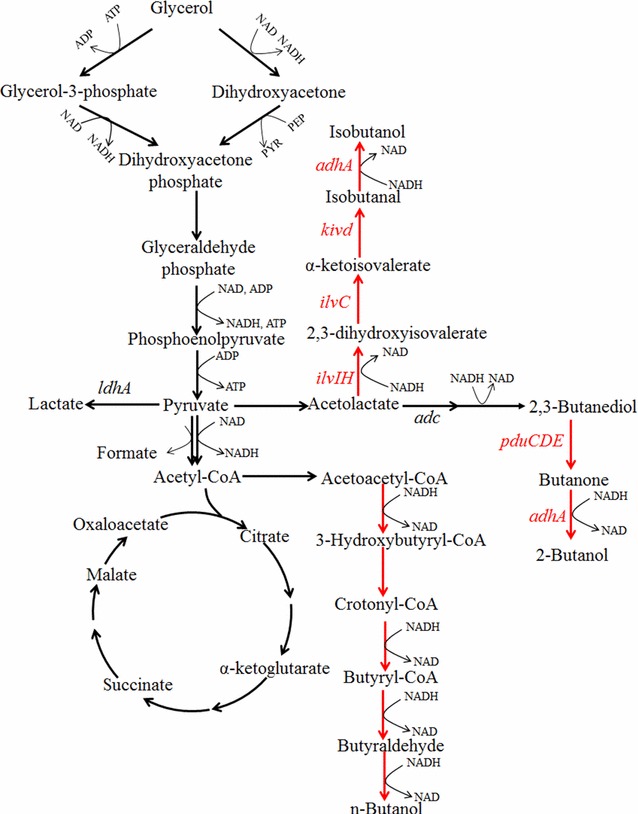
Metabolic pathway for the production of *n*-butanol, 2-butanol, and isobutanol. *adc* acetolactate decarboxylase; *adhA* alcohol dehydrogenase; *ilvC* keto-acid reducto-isomerase; *ilvIH* acetolactate synthase; *kivd* α-ketoisovalerate decarboxylase; *ldhA* lactate dehydrogenase; *pduCDE*: diol dehydratase

Butanol has received renewed interest recently as a potential green biofuel [77]. Compared to ethanol, butanol has higher energy density and lower hygroscopicity, and can be directly blended with gasoline without the need for modifying the current vehicle system. Production of butanol is a reducing equivalent-intensive process. Thus, glycerol can be utilized as a good substrate for butanol production. Three isoforms of butanol (*n*-butanol, 2-butanol, and isobutanol) can be used as biofuel and can be directly produced from glycerol. *Clostridium pasteurianum* can utilize crude glycerol to produce *n*-butanol and 1,3-PDO. To enhance the production of *n*-butanol, the *dhaT* gene was knocked out, resulting in an 83 % reduction of 1,3-PDO production [78]. The yield and selectivity (reported as g *n*-butanol produced per g of total solvents) of butanol was increased by 24 and 63 %. The *n*-butanol synthesis pathway has also been heterogeneously expressed in *E. coli* and *K. pneumoniae* (Fig. 3) [79, 80]. However, the currently reported titers were much lower in comparison to those using glucose as substrate [77]. More systematic strategies to optimize heterologous pathways and to reduce by-products formation should be applied to increase both the titer and yield. Isobutanol production from glycerol has also been achieved by expressing acetolactate synthase (*ilvIH*), keto-acid reducto-isomerase (*ilvC*), α-ketoisovalerate decarboxylase (*kivd*), and alcohol dehydrogenase (*adhA*) in *K. pneumoniae* (Fig. 3) [81]. By eliminating the lactate and 2,3-butanediol pathway (Δ*ldhA*Δ*adc*), the resulting strain can produce about 320 mg/L of isobutanol. 2-Butanol production from glycerol could be achieved via the extension of 2,3-BDO synthesis pathway with diol dehydratase (*pduCDE*) and alcohol dehydrogenase (*adh*) (Fig. 3) [41]. However, the activity of diol dehydratase toward *meso*-2,3-BDO dehydration should be significantly improved by protein engineering to get a higher titer of 2-butanol.

### Lipids and fatty acids

**Fig. 4:**
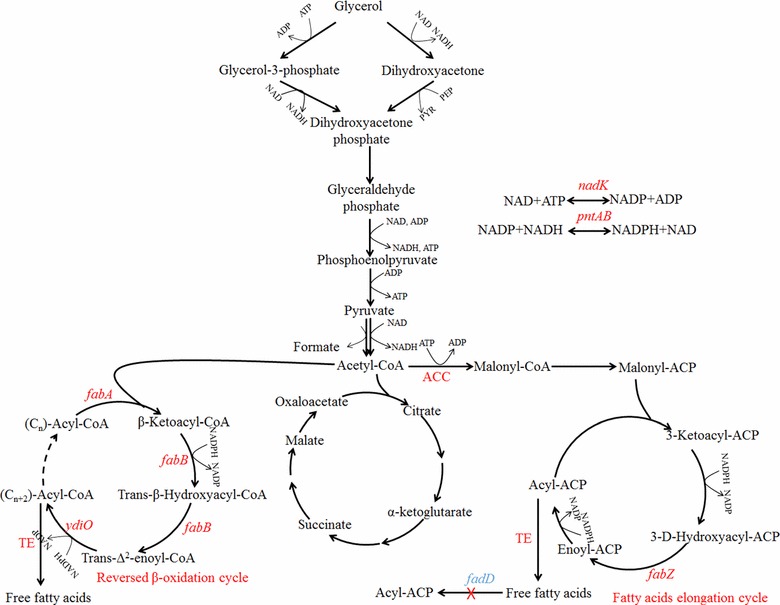
Metabolic pathway for the production of free fatty acids (FFAs) via fatty acid elongation cycle and reversed β-oxidation cycle. Strategies used to increase the yield of FFAs include (*1*) enhancement of precursor supply by overexpression of acetyl-CoA carboxylase (ACC); (*2*) repression of FFAs consumption via the deletion of acyl-CoA synthetase (*fadD*); (*3*) enhancement of fatty acid elongation cycle via the overexpression of (3R)-hydroxymyristoyl-ACP dehydrase (*fabZ*); (*4*) enhancement of NADPH generation via the overexpression of NAD kinase (*nadK*) and membrane-bound transhydrogenase (*pntAB*). *ACC* acetyl-CoA carboxylase; *fabZ*: (3R)-hydroxymyristoyl-ACP dehydrase; *fadA*, thiolase; *fadB*, functional hydroxyacyl-CoA dehydrogenase and enoyl-CoA hydratase; *fadD*: acyl-CoA synthetase; *nadK* NAD kinase; *pntAB* membrane-bound transhydrogenase; *TE* thioesterase; *ydiO*, enoyl-CoA reductase

Microbial lipids and free fatty acids (FFAs) are important sources for the production of biodiesel and other chemicals. A broad spectrum of microbes including microalgae, yeasts and fungi can efficiently utilize crude glycerol for the production of lipids [82–84]. Overexpression of glycerol utilization pathway such as glycerol kinase and glycerol 3-phosphate dehydrogenase was shown to be important for increasing lipid productivity in *Fistulifera solaris* [85] and *Synechocystis* sp. [86]. The theoretical yield of FFAs on glycerol is higher than that on glucose due to the higher reduction degree of glycerol [87]. To produce FFAs, effective acyl–acyl carrier protein (ACP) thioesterase (TE) should be introduced to release FFAs from acyl-ACP generated in fatty acid elongation cycle. Other important factors to increase the yield of FFAs include (1) enhancement of precursor supply (malonyl-CoA) by overexpression of acetyl-CoA carboxylase; (2) repression of FFAs consumption via the deletion of acyl-CoA synthetase (Δ*fadD*); (3) enhancement of fatty acid elongation cycle; (4) enhancement of NADPH generation (Fig. 4). The first two strategies were combined with the overexpression of a TE from *Cinnamomum camphora* by Lu et al. who constructed a mutant *E. coli* strain that can accumulate FFAs up to 2.5 g/L from glycerol [88]. Similar strategies were adopted by Lenne et al. who further optimized the copy number of TE from *Umbellularia californica* [89]. A lower copy of TE was shown to be more beneficial for FFAs production. To enhance the supply of NADPH for FFAs production, overexpression of both NAD kinase (*nadK*) and membrane-bound transhydrogenase (*pntAB*) was shown to be an effective strategy to enhance the production and yields of FFAs (4.82 g/L and 0.3 g/g) [90]. Production of FFAs with varied chain length from glycerol by the engineered reversal of β-oxidation cycle was also reported [91]. The system contained thiolase (*fadA*), 3-hydroxyacyl-CoA dehydrogenase (*fadB*), enoyl-CoA hydratase (*fadB*), enoyl-CoA reductase (*yidO*), and thioesterases (TE) (Fig. 4). Thioesterases could be used to control the chain length of FFAs.

### Challenges and future prospects

Biorefineries are based on the integration of various conversion processes to fully utilize biomass for the production of energy, fuels, and chemicals. In this context, the utilization of crude glycerol generated in the biodiesel and bioethanol production processes to produce valuable chemicals and fuels offers an excellent opportunity to improve resource efficiency and economic viability of the biofuel industry. So far, most of the glycerol transformation processes are based on the fermentation of refined glycerol. The performance of the engineered strains on crude glycerol should be further examined. The composition of crude glycerol varies significantly, depending on the feedstock, the process used for biodiesel production, and the post-treatment methods. The impurities of crude glycerol include water, methanol, salts, soap, fatty acids, and other unknown substances [2, 8]. Some of the impurities may significantly affect the performance of microorganisms. For example, it was reported that only three of the seven tested samples can be utilized by a metabolic engineered *C. glutamicum* for amino acids production [71]. Thus, it is important to elucidate the inhibition mechanism and enhance the tolerance of engineered strains to inhibitory substances. To this end, metabolic engineered strains can be further evolved to adapt to different glycerol lots by adaptive laboratory evolution and systems biology can be utilized to understand how specific mutations affect cellular metabolism. Moreover, systems biology can be utilized to identify novel targets which cannot be found by rational analysis for designing more efficient strains [15, 17, 18].

Recent development of synthetic biology can also be utilized to develop strains for the production of natural and non-natural products from glycerol. For example, novel metabolic pathways have recently been developed to produce ethylene glycol [13] and 1,4-butanediol [92], two most widely used diols in the polymer industry. Production of highly valuable pharmaceuticals such as artemisinin can also be implemented in glycerol metabolic pathway, providing new potentials for glycerol biorefinery [5, 93].

## Conclusions

The use of metabolic engineering to improve the performance of industrial strain for converting abundant and low-priced crude glycerol into higher value products represents a promising route toward glycerol biorefinery, which will significantly increase the economic viability of the biofuels industry. Current development of systems biology, synthetic biology, and new tools of metabolic engineering need to be integrated to improve the process efficiency (titer, yield and productivity) and to produce new products such as non-natural chemicals. Efforts also need to be paid to engineer and adapt strains for industrial implementation, especially for enhanced tolerance to toxic substances of crude glycerol and variable industrial environments.

## Abbreviations

DHA: dihydroxyacetone
1,3-PDO: 1,3-propanediol
1,2-PDO: 1,2-propanediol
ACP: acyl–acyl carrier protein
TE: thioesterase
PEPC: phosphoenolpyruvate carboxylase
PEPCK: phosphoenolpyruvate carboxykinase
PYC: pyruvate carboxylase
3-HP: 3-hydroxypropionic acid
